# Importance of extracellular matrix and growth state for the EA.hy926 endothelial cell response to polyunsaturated fatty acids

**DOI:** 10.1371/journal.pone.0197613

**Published:** 2018-05-15

**Authors:** Youjia Du, Carla G. Taylor, Harold M. Aukema, Peter Zahradka

**Affiliations:** 1 Department of Physiology & Pathophysiology, University of Manitoba, Winnipeg, Manitoba, Canada; 2 Canadian Centre for Agri-Food Research in Health and Medicine, St. Boniface Albrechtsen Research, Winnipeg, Manitoba, Canada; 3 Department of Food and Human Nutritional Sciences, University of Manitoba, Winnipeg, Manitoba, Canada; Southern Illinois University School of Medicine, UNITED STATES

## Abstract

Consumption of different PUFAs (polyunsaturated fatty acids) can induce functional changes in blood vessels via endothelial cells, which interact with dietary factors in the circulation. The basement membrane that separates the endothelium from the smooth muscle cells of the medial layer can also influence the functional state of endothelial cells. However, the effect of basement membrane on the endothelial response to dietary PUFAs in relation to growth state (e.g. proliferation versus quiescence) has never been investigated. We therefore compared the viability (CCK kit) and proliferation (bromodeoxyuridine incorporation) of EA.hy926 endothelial cells grown on Matrigel or collagen versus non-coated plates. EA.hy926 viability and proliferation were also assessed after treatment with 0–150 μM of PUFAs [linoleic acid (LA), arachidonic acid (AA), α-linolenic acid (ALA), eicosapentaenoic acid (EPA) and docosahexaenoic acid (DHA)]. Our study showed that only cells grown on Matrigel-coated plates reached quiescence after becoming confluent with a decreased level of MCM2 and p-cyclin D1 (T286), increased levels of p27kip1 and a low level of apoptosis and senescence. AA, EPA and DHA decreased the viability and proliferation of subconfluent cells grown on plastic dishes in a dose-dependent manner, while the presence of Matrigel made the cells resistant to these adverse effects. Confluent cell viability was less sensitive to higher concentrations of AA, EPA and DHA than subconfluent cells, and a significant increase in caspase-3 cleavage was only observed in confluent cells treated with DHA. Higher concentrations of AA, EPA and DHA suppressed DNA synthesis by both subconfluent and confluent cells, while precursor C18 PUFAs (LA and ALA) had no negative effects on viability and proliferation. Our study is the first to show that extracellular matrix and growth state are important factors in the EA.hy926 cell response to PUFAs, and that the mechanisms by which individual PUFAs operate may be growth state-dependent.

## Introduction

The endothelium is formed by a single layer of endothelial cells and lines the luminal surface of all blood vessels. Healthy endothelial cells play an important role in maintaining vascular homeostasis [1], and function as a barrier that is selectively permeable for the transportation of key molecules. Endothelial cell proliferation and migration also contribute to the formation of blood vessels in response to the influence of various hormones. Endothelial cells regulate vascular tone by secreting vasodilators such as nitric oxide and prostacyclin I_2,_ and vasoconstrictors such as endothelin. Endothelial cells are responsible for creating the anti-thrombotic and anti-coagulant environment required for the circulation. Moreover, endothelial cells participate in inflammatory responses by expressing adhesion molecules, as well as metalloproteinases. Endothelial cells are attached to the basement membrane, an extracellular matrix (ECM) protein-rich layer located beneath the endothelium that provides physical and chemical support. Adhesion of endothelial cells to basement membrane proteins through integrins is important for maintaining endothelial cell viability and strongly influences other functions such as proliferation, migration, vessel formation and blood vessel stabilization [2].

In plasma, total non-esterified fatty acid (FA) levels can reach 300 μM in healthy individuals and higher in persons with type 2 diabetes [3]. At these concentrations, FAs could have a major influence on vascular physiology. Based on dietary intervention studies that examined how modulation of endothelial function by different dietary polyunsaturated fatty acids (PUFAs) affects vascular function, it is believed that incorporating n-3 PUFA into the diet improves endothelial function as indicated by flow-mediated dilatation [4–7]. However, the mechanism of action for PUFA, particularly in relation to specific endothelial processes, has received limited attention.

The functional properties of endothelial cells are strongly influenced by the substrate on which they grow [8–10]. Dishes coated with ECM proteins such as collagen, gelatin and laminin are used for culturing adherent cells such as endothelial cells. Matrigel, which is composed of a mixture of ECM proteins derived from tumor cells, is an alternative substrate. Interestingly, little attention has been paid to how ECM influences the viability and proliferation of endothelial cells, which are critical factors contributing to the role of endothelial cells in normal vascular physiology as well as pathophysiology. In the healthy endothelium, endothelial cells are confluent and non-proliferating. In contrast, endothelial cells must proliferate when the endothelium has been damaged and is under repair. It is recognized that certain dietary factors can damage the endothelium, but interestingly, fats are associated with both positive and negative effects on the endothelium. However, the effect of PUFAs on the viability and proliferation of endothelial cells in the context of both ECM substrate and growth state has not been investigated.

To address these concepts, the current study investigated how EA.hy926 endothelial cells, a fusion of human umbilical vein endothelial cells with human carcinoma A549 cells [11], respond to different n-6 [linoleic acid (LA) and arachidonic acid (AA)] and n-3 [α-linolenic acid (ALA), eicosapentaenoic acid (EPA) and docosahexaenoic acid (DHA)] PUFAs in terms of viability and proliferation when they are grown on different ECM substrates. The response of EA.hy926 endothelial cells to PUFAs in different growth states (i.e., growing and confluent) was considered in relation to proteins involved in cell viability and proliferation as a means of investigating their mechanisms of action.

## Materials and methods

### Cell culture

EA.hy926 endothelial cells were purchased from ATCC (cat. CRL 2922, Manassas, Virginia, USA) and propagated according to the manufacturer’s protocol. EA.hy926 cells were cultured in Dulbecco’s modified Eagle’s medium (DMEM) with 20 mM HEPES, 100 units/mL penicillin, and 100 μg/mL streptomycin supplemented with 10% fetal bovine serum (FBS). The cells were maintained in a humidified 5% CO_2_ atmosphere at 37°C. Cells were used within 20 passages.

### ECM coatings

Growth Factor Reduced Matrigel (Corning®, Product No 356231) was handled according to the manufacturer’s protocol. Each well of the 96-well plate received 100 μL of DMEM containing 5 μL of Matrigel. The plates were left for 1 hour at room temperature and were washed once with DMEM before seeding the cells. Collagen Type I (Sigma, Product No. C7661) was dissolved in acetic acid according to the manufacturer’s protocol, and concentrations of 6 and 10 μg/cm^2^ (manufacturer’s suggested lower and upper concentrations) were used to coat the dishes for 5 hours at 37°C after which the liquid was removed and the well allowed to dry overnight under a UV light. The coated surface was rinsed with phosphate-buffered saline (PBS; 137 mM NaCl, 2.7 mM KCl, 10 mM Na_2_HPO_4_, 1.8 mM KH_2_PO_4_) before cell seeding.

### Cell preparation

EA.hy926 endothelial cells were seeded at a density of 9000 cells/cm^2^. The day cells were seeded was referred to as day 0. Subconfluent cells were used at day 4, and confluent cells were used at day 8. EA.hy926 endothelial cells were grown on Matrigel or collagen or non-coated plates for up to 12 days.

### Polyunsaturated fatty acid (PUFA) treatment

Polyunsaturated fatty acids (LA, AA, ALA, EPA and DHA; Cayman Chemical) were dissolved in ethanol and then mixed with 5% fatty acid free bovine serum albumin in PBS. They were added directly to the cell culture medium without disturbing the cells.

### Cell viability

Cell viability (total cell number) was assessed with the CCK-8 (cell counting kit, Dojindo Molec Tech) assay. CCK reagent was added to the cells and incubated for 90 min. The absorbance of the media was measured at 450 nm using a microplate reader.

### Cell proliferation

Proliferation of cells was measured with the Bromodeoxyuridine (BrdU) Cell Proliferation kit (Millipore). BrdU reagent was added to the cells and incubated for 4 h. Cells were fixed, DNA was denatured, and incorporated BrdU was detected immunochemically according to the kit instructions. Plates were measured at a dual wavelength of 450/550 nm.

### Western blotting

Protein was extracted from EA.hy926 cells grown on Matrigel-coated and plastic culture plates. Cells were lysed each day (day 4 to day 12) with 2× sample buffer (125 mM Tris-HCl pH 6.8, 2% Sodium dodecyl sulphate, 20% glycerol) and briefly sonicated. Subconfluent and confluent EA.hy926 cells grown on Matrigel-coated plates were also lysed and sonicated after treatment with 125 μM of PUFAs (LA, AA, ALA, EPA and DHA) for 8 h. Twenty μg of lysate protein were subjected to SDS-polyacrylamide gel electrophoresis, then transferred electrophoretically to polyvinylidene difluoride (PVDF) membranes for immunoblotting. Primary antibodies were diluted in TBST (Tris-buffered saline with Tween-20; 150 mM NaCl, 20 mM Tris-HCl pH 7.4, 0.05% Tween 20) containing 3% bovine serum albumin (BSA). The primary antibodies employed were rabbit anti-MCM2 (1:1000, abcam, #4007), rabbit anti-phospho-cyclin D1 (Thr286) (1:1000, Cell Signaling, #3300), mouse anti-cyclin D1 (1:1000, Cell Signaling, #2926), rabbit anti-VE (vascular endothelial)-cadherin (1:1000, Cell Signaling, #2500), rabbit-anti-p27 kip1 (1:1000, Cell Signaling, #3686), rabbit anti-cleaved caspase-3 (1:1000, Cell Signaling, #9664) and rabbit anti-caspase-3 (1:1000, Cell Signaling, #9662). Horseradish peroxidase (HRP) conjugated secondary antibodies were used at a dilution of 1:10000 in TBST containing 1% BSA. The relative band intensities were captured using a FluorChem® Q imager and quantified with AlphaView software (Alpha Innotech). The Western blot data were normalized to a Ponceau S stained image of the blot.

### Senescence β-galactosidase staining

Senescence was detected using the Senescence β-Galactosidase Staining Kit (Cell Signaling, #9860). EA.hy926 cells were rinse with PBS, then fixed and stained with X-Gal according to the manufacturer’s protocol. Cells were incubated in a 37°C incubator (no CO_2_) overnight and imaged under a light microscope (200× magnification). X Gal was stained blue when cleaved by β-galactosidase.

### Statistical analysis

One-way ANOVA and Tukey’s test were used to assess statistical significance, which was set at p<0.05 (SPSS version 20). All experiments were independently repeated at least 3 times. All values are expressed as mean ± SEM (n = 3).

## Results

### Effects of basement membrane

**Fig 1:**
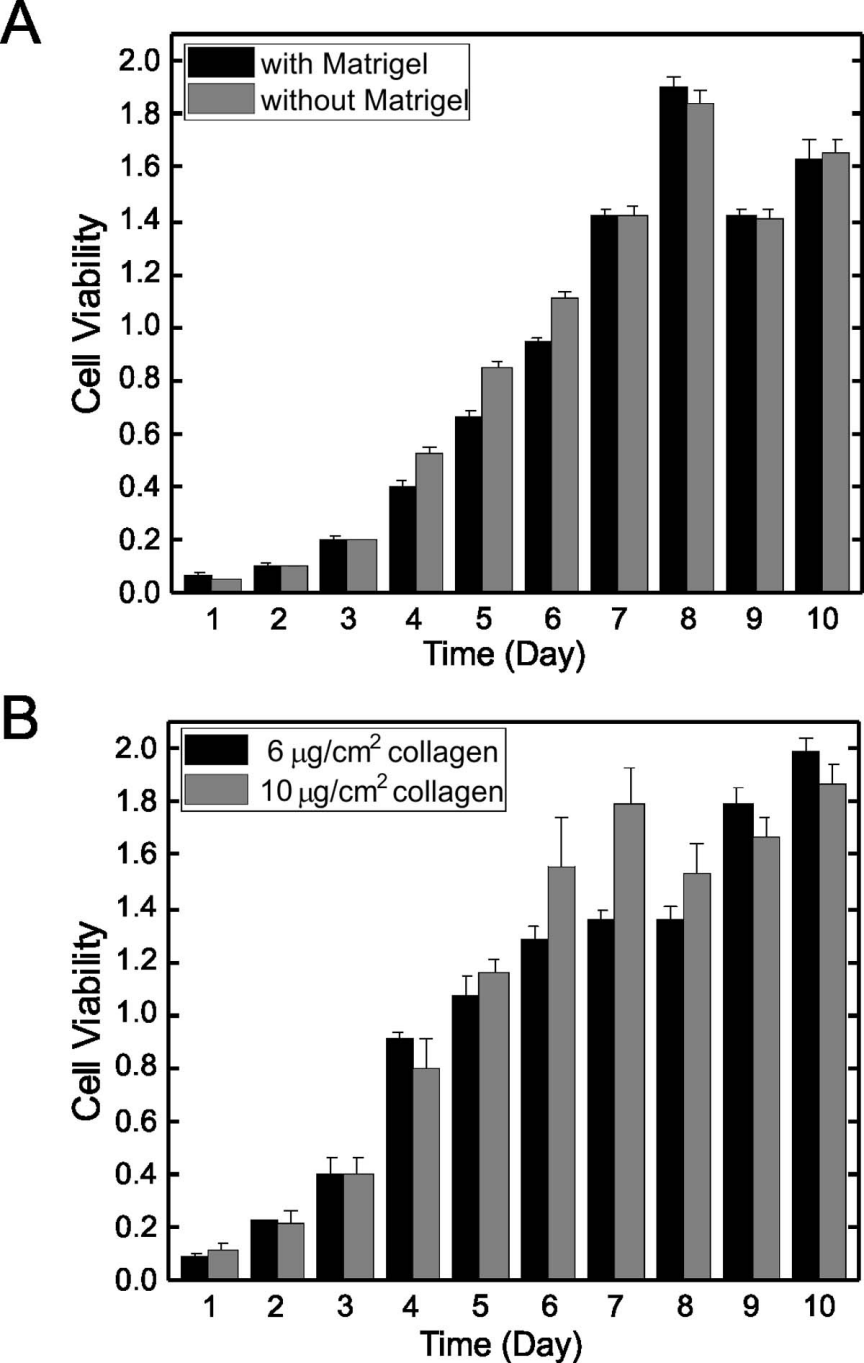
Effect of ECM substrate on growth of EA.hy926 cells. EA.hy926 endothelial cells were seeded at 9000 cells/cm^2^ on 96 well plates pre-coated with Matrigel (5 μL Matrigel mixed in 95 μL DMEM) or without Matrigel (Panel A) or Collagen Type I dissolved in acetic acid at concentrations of 6 and 10 μg/cm^2^ (Panel B) and grown for up to 10 days without changing the media. Cell number was determined with the CCK-8 assay. A microplate reader was used to read absorbance of the media at 450 nm. Data are plotted as means ± SEM (n = 3).

Growth of endothelial cells with and without Matrigel had no significant effect on viability or total cell number (Fig 1A). Cells reached a maximal number at day 8. This was defined as confluence and confirmed visually. Similarly, no significant differences in cell viability or total cell number were observed when cells were grown on collagen-coated plates, at two different concentrations of collagen (Fig 1B).

**Fig 2:**
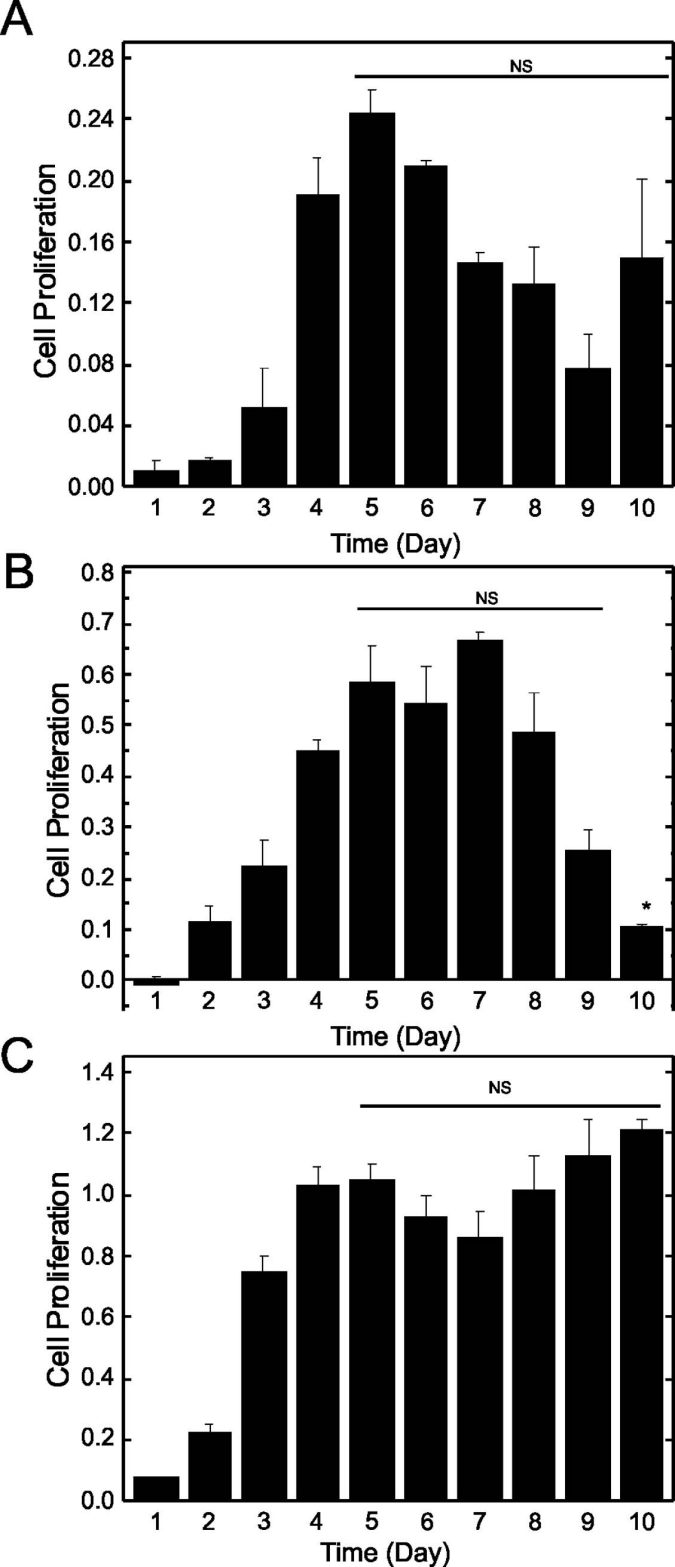
Effect of ECM substrate on proliferation of EA.hy926 cells. EA.hy926 endothelial cells were seeded at a density of 9000 cells/cm^2^ on non-coated (Panel A), Matrigel-coated (Panel B) or collagen-coated (Panel C) 96 well plates and grown for up to 10 days without changing the media. Cell proliferation was assessed with the BrdU cell proliferation assay. Plates were read at dual wavelength 450/550 nm. Data are plotted as means ± SEM (n = 3). NS, non-significant versus day 4; * significantly different (p <0.05) from day 4.

When EA.hy926 endothelial cells were grown on non-coated plates, the DNA synthesis rate peaked on day 5, but DNA synthesis remained elevated to day 10 (Fig 2A) even though the cells visually reached confluence at day 8. When EA.hy926 endothelial cells were grown on Matrigel, the DNA synthesis rate reached a maximum on day 4 and then started to decrease after day 8 (Fig 2B), suggesting that the cells became quiescent after reaching confluence (day 8) on Matrigel-coated plates. However, when EA.hy926 endothelial cells were grown on collagen coated plates, the DNA synthesis rate remained high from day 4 to day 10 (Fig 2C), similar to cells grown without basement membrane. Thus, subsequent experiments focused on cells grown on plastic plates with and without Matrigel as the substrate.

**Fig 3:**
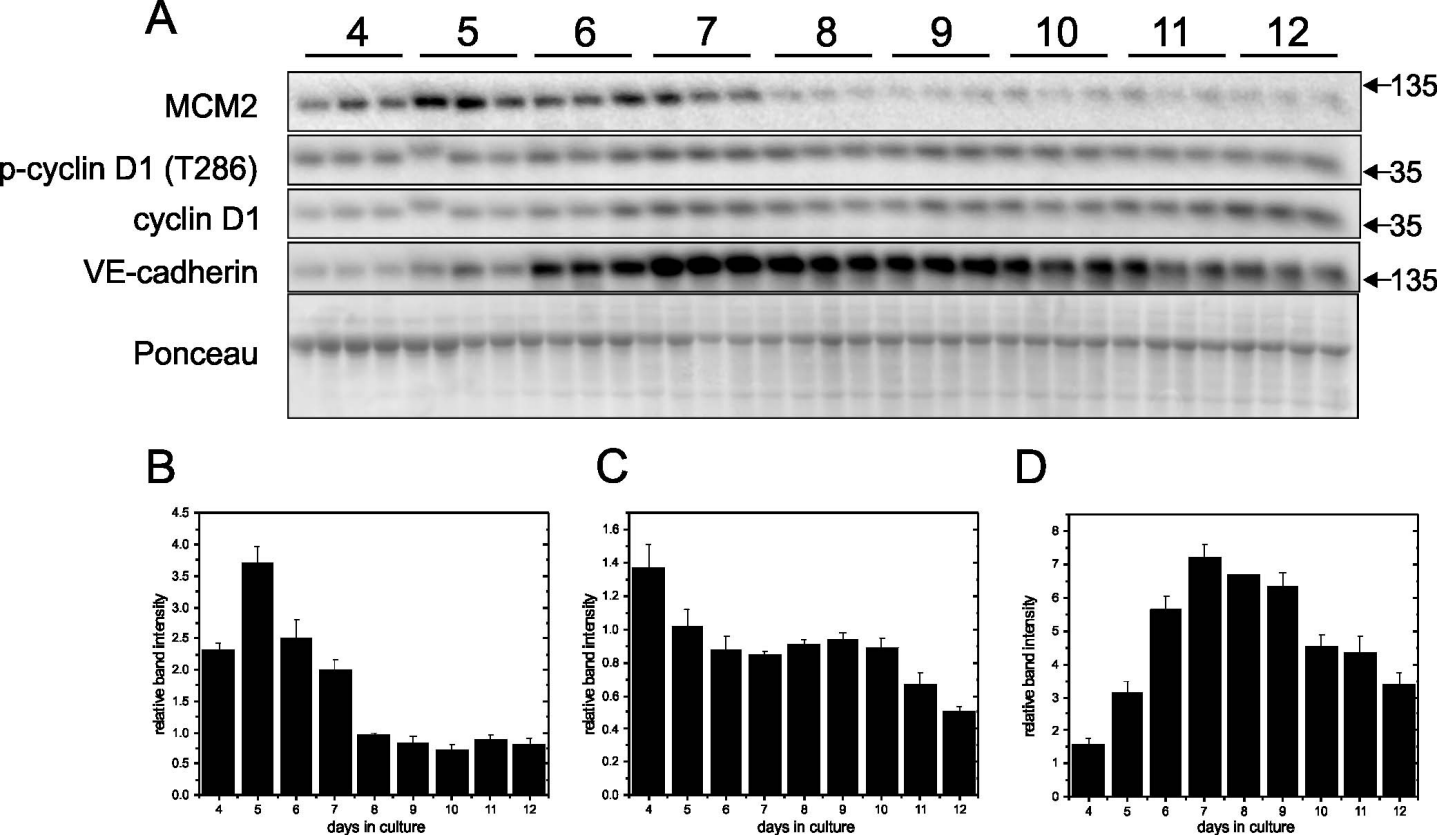
Time course for MCM2, p-cyclinD1, cyclin D1 and VE-cadherin in EA.hy926 cells grown on Matrigel-coated plates. EA.hy926 endothelial cells were seeded on Matrigel-coated plates at 9000 cells/cm^2^ and grown for 12 days. Protein levels of MCM2, p-cyclinD1, cyclin D1 and VE-cadherin were determined by Western blotting of cell lysates prepared each day from day 4 to day 12. Representative blots are shown in Panel A. Densitometry was used to quantify the intensity of the bands in the upper panel and data were normalized to a band visualized by Ponceau staining. Data are presented as means ± SEM (n = 3) for MCM2 (Panel B), p-cyclin D1/cyclin D1 (Panel C) and VE-cadherin (Panel D).

**Fig 4:**
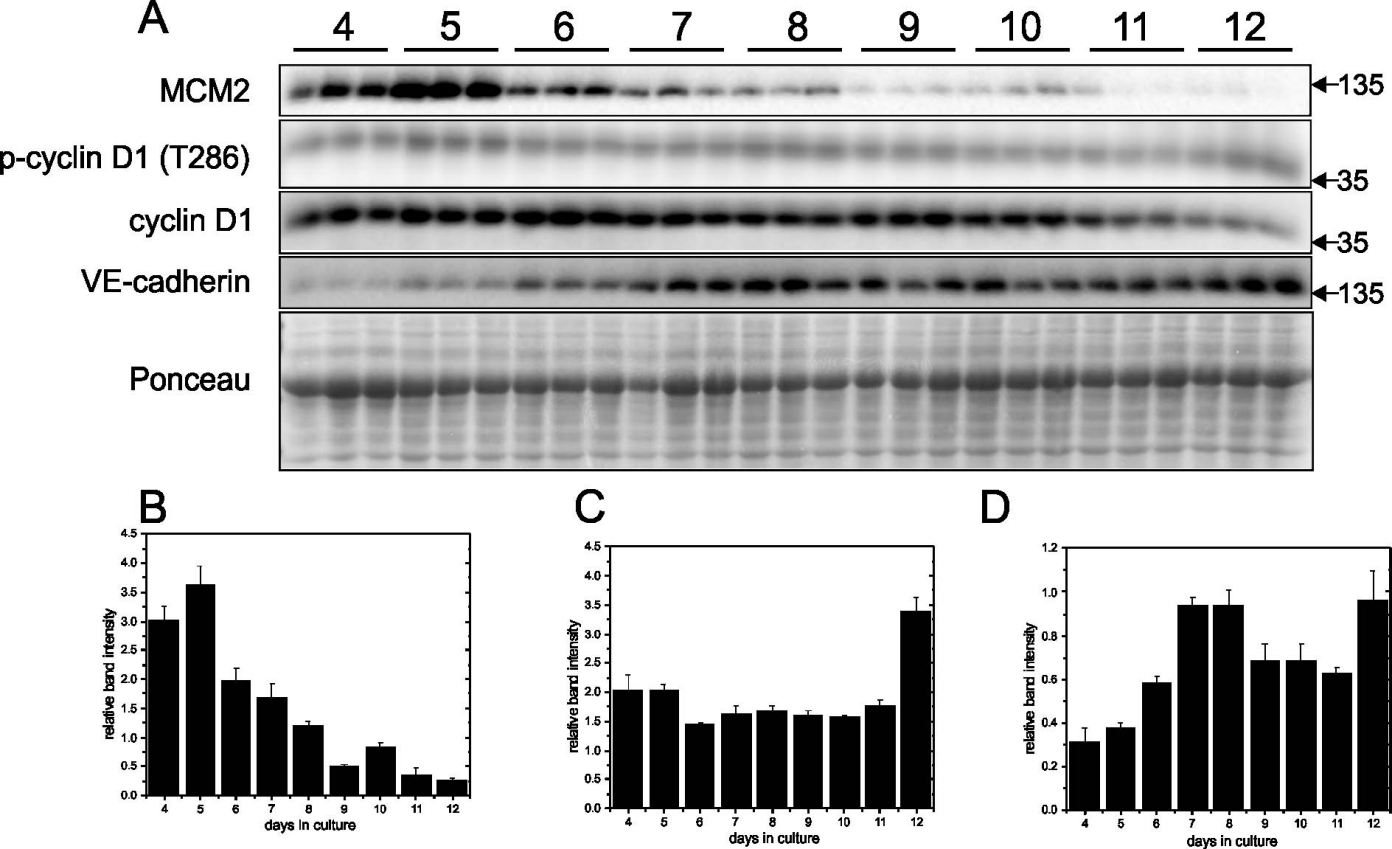
Time course for MCM2, p-cyclinD1, cyclin D1 and VE-cadherin in EA.hy926 cells grown on plastic culture plates. EA.hy926 endothelial cells were seeded on plastic plates at 9000 cells/cm^2^ and grown for 12 days. Protein levels of MCM2, p-cyclinD1, cyclin D1 and VE-cadherin were determined by Western blotting of cell lysates prepared each day from day 4 to day 12. Representative blots are shown in Panel A. Densitometry was used to quantify the intensity of the bands in the upper panel and data were normalized to a band visualized by Ponceau staining. Data are presented as means ± SEM (n = 3) for MCM2 (Panel B), p-cyclin D1/cyclin D1 (Panel C) and VE-cadherin (Panel D).

To verify the DNA synthesis measurements, Western Blotting was used to examine expression of MCM2, p-cyclin D1 (T286), cyclin D1 and VE-cadherin in cells grown on Matrigel-coated and plastic plates. MCM2 is a key component of the MCM2-7 complex that is essential for DNA replication initiation and elongation in eukaryotic cells. MCM2 peaked at day 5 in cells grown on Matrigel-coated plates and started to decline afterwards, and this suggested the cells were entering a quiescent state after reaching confluence (Fig 3B). This interpretation was tested with p-cyclin D1, an important regulator in cell cycle progression that contributes to the entry from G_1_ to S phase. Since phosphorylation at T286 facilitates cyclin D1 nuclear export to the cytoplasm for degradation and thus progression through the cell cycle, a decline of the p-cyclin D1 (T286)/cyclin D1 ratio (Fig 3C) in the confluent cells was considered an indication of a decrease in actively cycling cells. VE-cadherin, which is endothelial specific and belongs to the adherens junction family, peaked on day 7 (Fig 3D). The presence of VE-cadherin indicated intercellular junctions had formed, thus confirming the EA.hy926 cells were a confluent monolayer. In contrast, when cells were grown on plastic plates, the p-cyclin D1 (T286)/cyclin D1 ratio remained high until day 12 when it reached its greatest elevation (Fig 4C). These results indicate that the cells were not entering a quiescent state even if MCM2 levels had declined (Fig 4B). The VE-cadherin level was also high (Fig 4D) suggesting that cell-cell contacts were continuously present.

**Fig 5:**
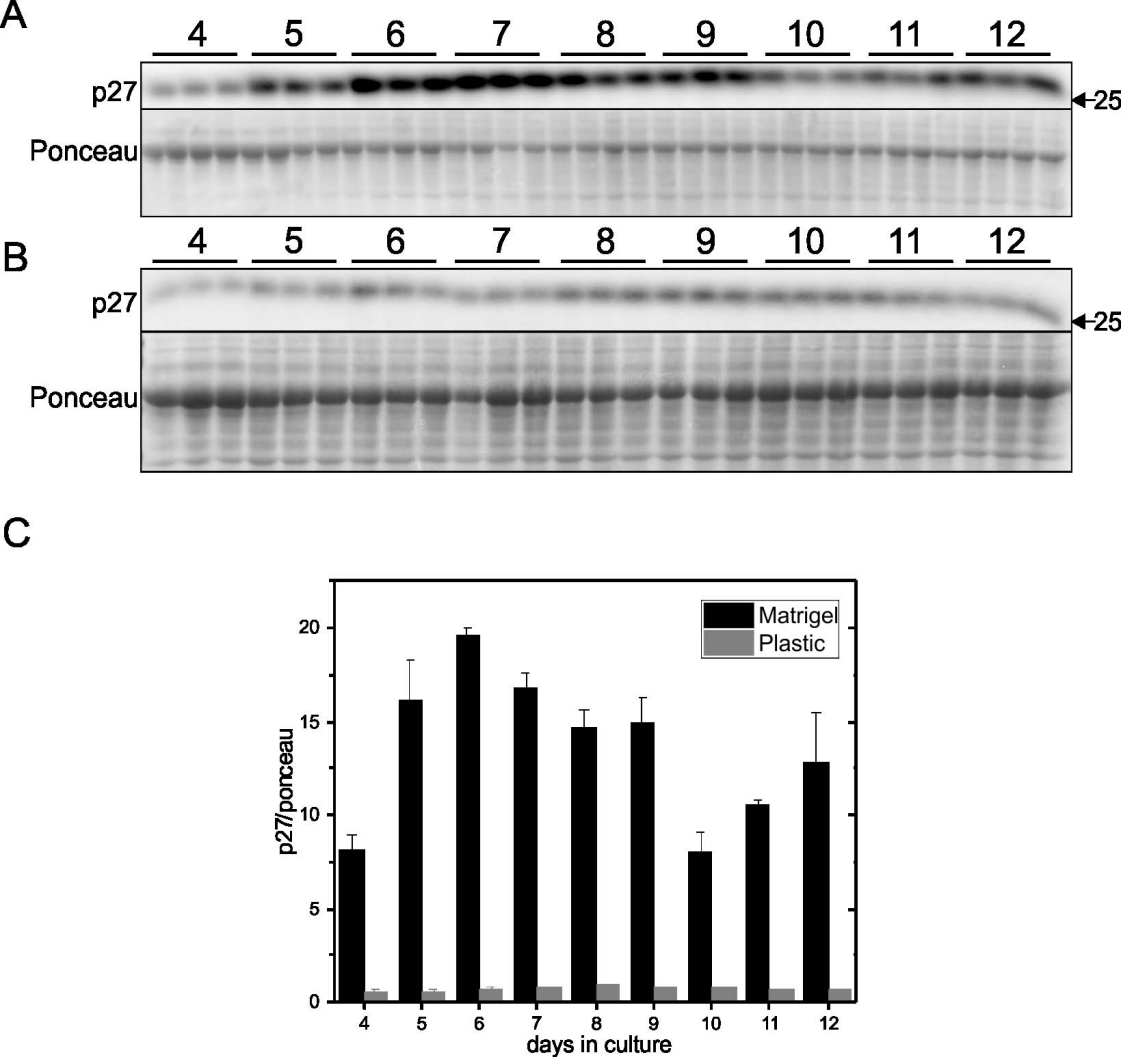
Time course of p27kip1 in EA.hy926 cells grown on Matrigel-coated or plastic culture plates. EA.hy926 endothelial cells were seeded on plastic plates at 9000 cells/cm^2^ and grown for 12 days. Protein levels of p27kip1 were determined by Western blotting of cell lysates prepared each day from day 4 to day 12. Representative blots are shown in Panel A (Matrigel) and B (Plastic). Densitometry was used to quantify the intensity of the bands in Panel A and B, and data were normalized to a band visualized by Ponceau staining (Panel C). Data are presented as means ± SEM (n = 3).

**Fig 6:**
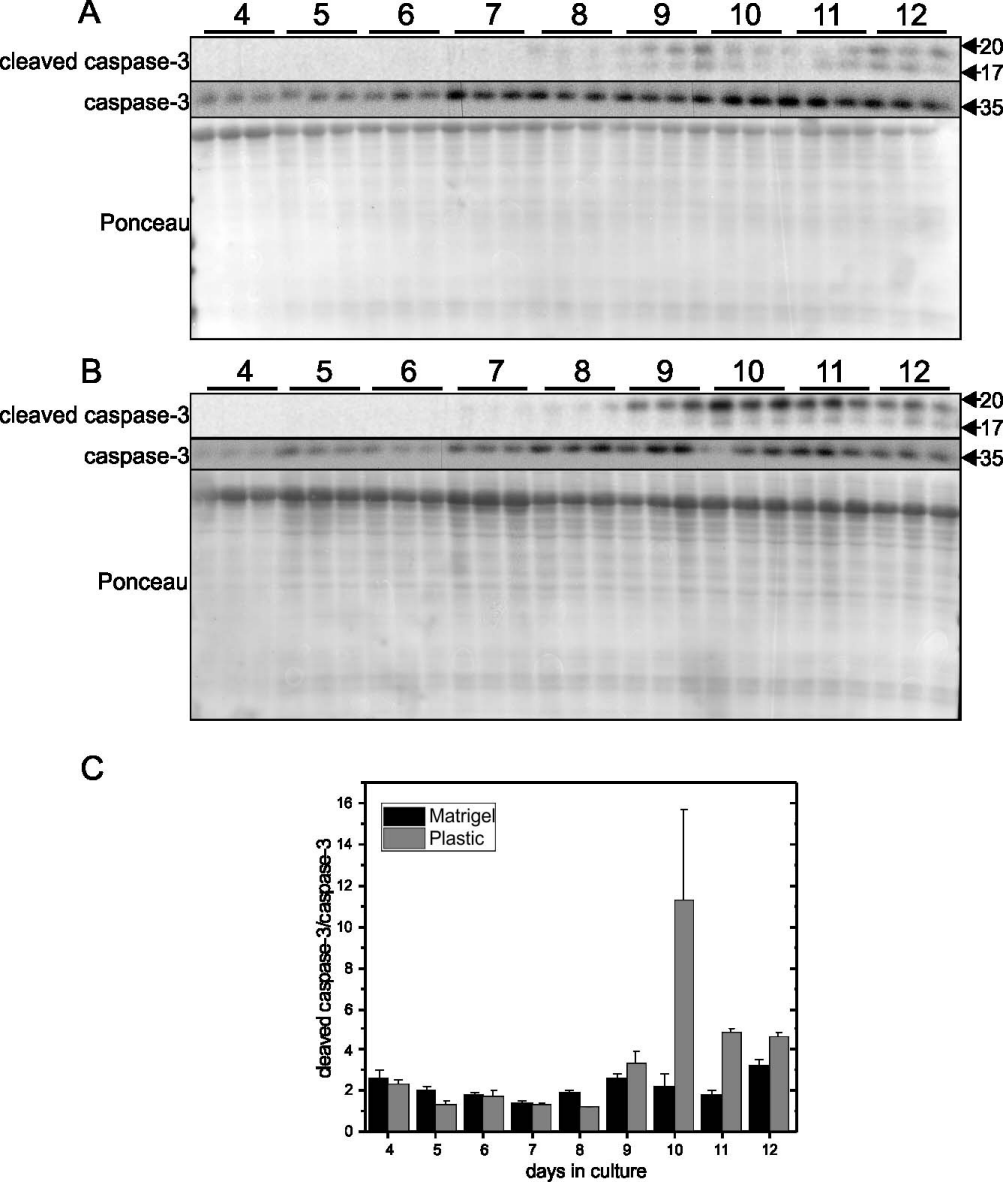
Time course of cleaved caspase-3 and caspase-3 in EA.hy926 cells grown on Matrigel-coated or plastic culture plates. EA.hy926 endothelial cells were seeded on plastic plates at 9000 cells/cm^2^ and grown for 12 days. Protein levels of cleaved caspase-3 and caspase-3 were determined by Western blotting of cell lysates prepared each day from day 4 to day 12. Representative blots are shown in Panel A (Matrigel) and B (Plastic). Densitometry was used to quantify the intensity of the bands in Panel A and B, and data were normalized to a band visualized by Ponceau staining (Panel C). Data are presented as means ± SEM (n = 3).

**Fig 7:**
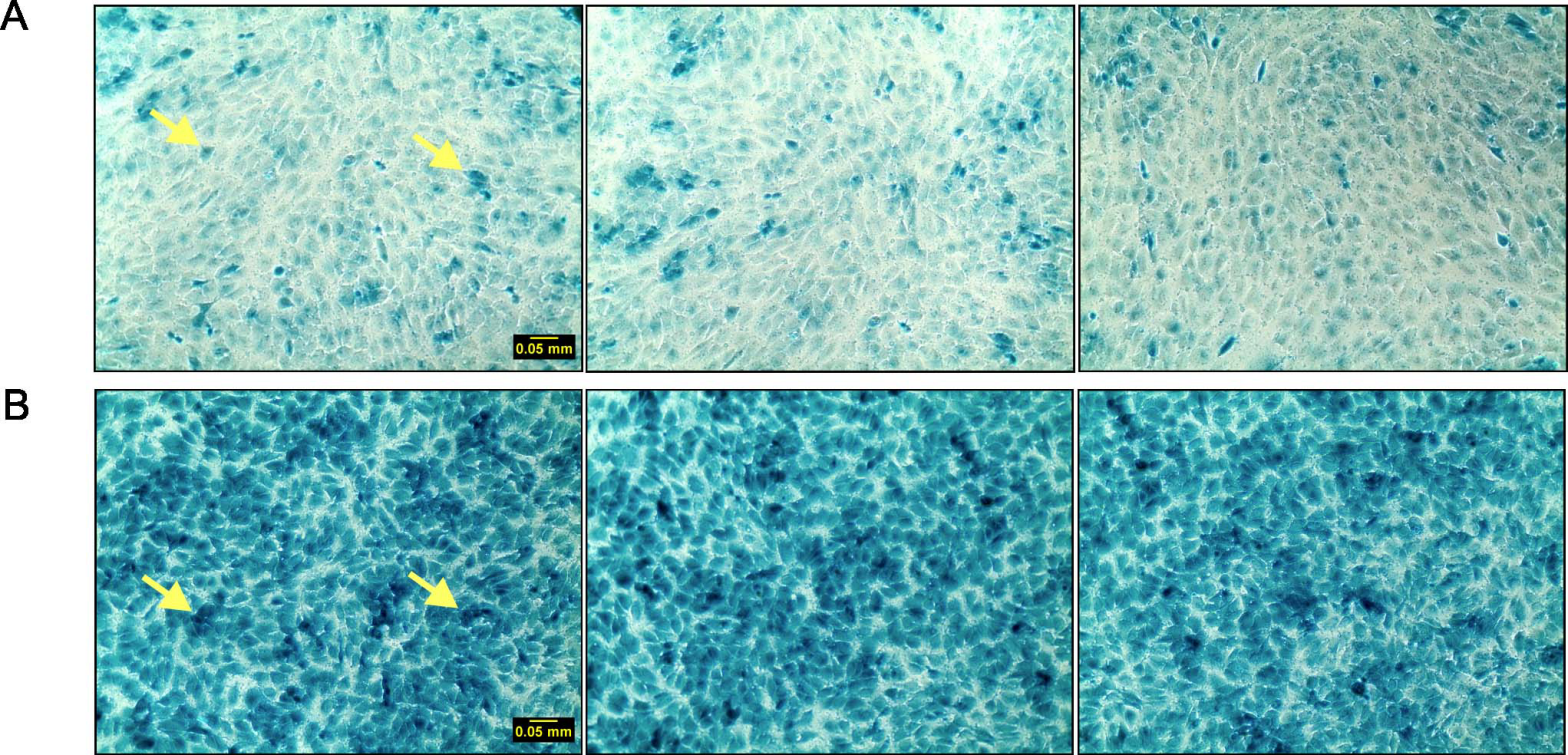
Senescence-associated β-galactosidase staining in EA.hy926 cells grown on Matrigel-coated or plastic culture plates. EA.hy926 endothelial cells were seeded on Matrigel-coated (Panel A) or plastic plates (Panel B) at 9000 cells/cm^2^ and grown for 10 days. SA-β-Galactosidase Staining was performed on cells and 3 representative images are shown per condition. The arrows in the first image of Panel A and Panel B indicate cells with a high level of SA-β-galactosidase staining.

To confirm the conclusion that cells grown on Matrigel enter a quiescent state after reaching confluence, expression of p27kip1, cleaved caspase-3 and caspase-3, and β-galactosidase activity were examined in cells grown on Matrigel-coated or plastic plates. p27kip1 is an important negative regulator of cyclin-dependent kinase (CDK) and limits cell cycle progression from G1 to S phase. The higher levels of p27kip1 in cells grown on Matrigel-coated plates after day 4 (Fig 5A) compared to cells grown on plastic plates (Fig 5B) demonstrated that cell cycle progression has been greatly limited in cells with Matrigel as the substrate (Fig 5). Caspase-3 activation is a key mediator and hallmark of apoptosis. The ratio of cleaved caspase-3/caspase-3 peaked on day 10 and continued at high levels in cells grown on plastic plates (Fig 6B), while the ratio in cells grown on Matrigel (Fig 6A) was relatively lower. Senescence-associated (SA) β-galactosidase activity, which is specifically detected at pH 6.0, is a widely used biomarker of cell senescence [12]. Fig 7 shows that there were more senescent cells present in cultures grown on plastic plates (Fig 7B) than in cultures grown on Matrigel-coated plates (Fig 7A). This finding indicates that cells grown on plastic have greater potential to become senescent, while cells grown on Matrigel-coated plates are less likely to be senescent. Therefore, confluency appears to promote entry of cells into a quiescent stage characterized by low levels of DNA synthesis and SA-β-galactosidase.

### Effects of PUFAs

**Fig 8:**
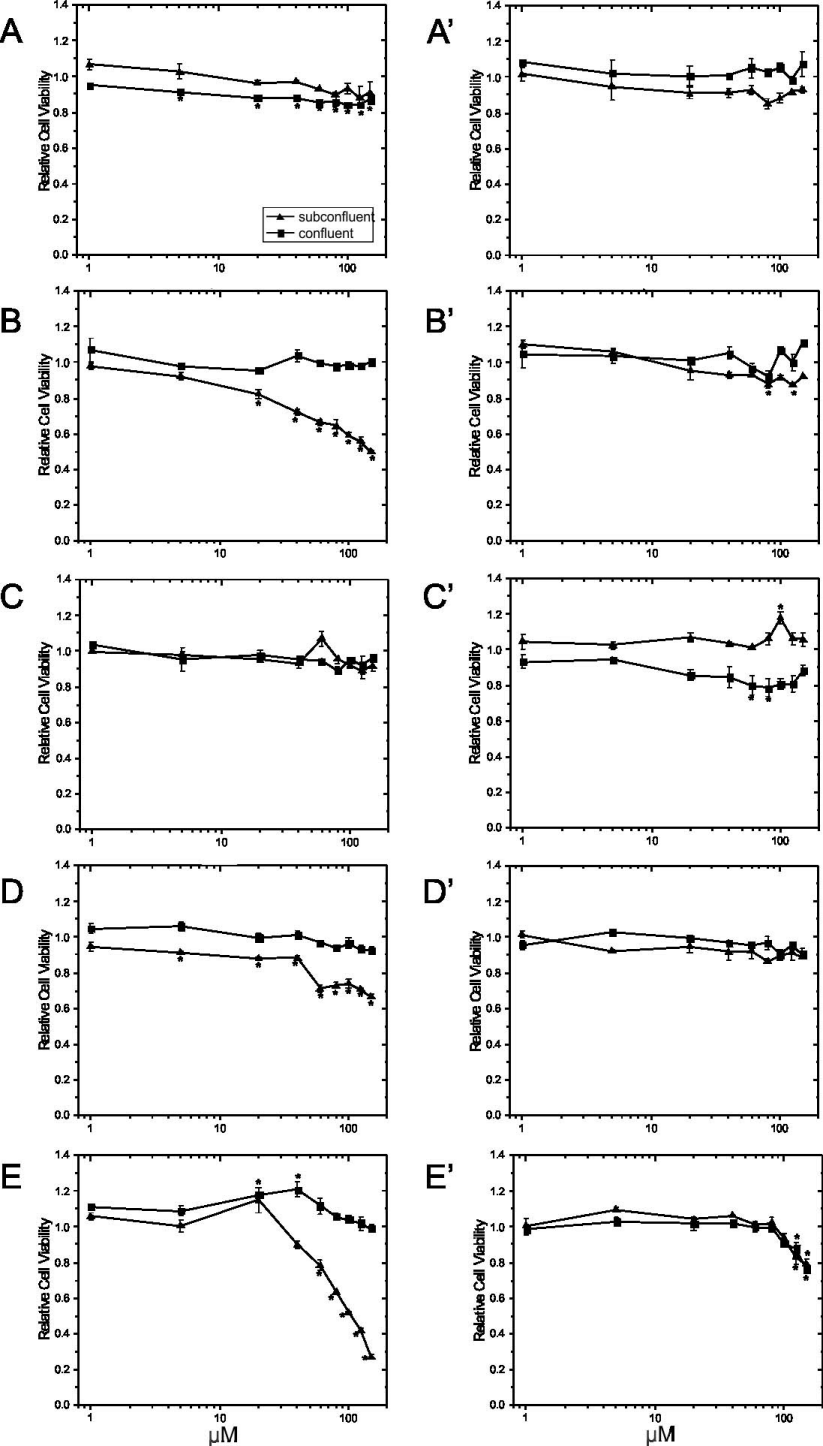
Effect of ECM substrate and growth state on viability of EA.hy926 cells in presence of PUFAs. EA.hy926 endothelial cells were seeded on plastic culture plates at 9000 cells/cm^2^ in the absence (Panel A-E) or presence of Matrigel (Panel A’-E’). Growing cells (day 4) or confluent cells (day 8) were treated with different PUFAs [LA (Panel A, A’), AA (Panel B, B’), ALA (Panel C, C’), EPA (Panel D, D’) and DHA (Panel E, E’)] at final concentrations of 1, 5, 20, 40, 60, 80, 100, 125 and 150 μM for 24 hours, at which point, cell viability was assessed using the CCK-8 assay. Data are normalized with vehicle control values and plotted as means ± SEM (n = 3). *Significantly different (p <0.05) from the vehicle control for the respective growth state.

**Fig 9:**
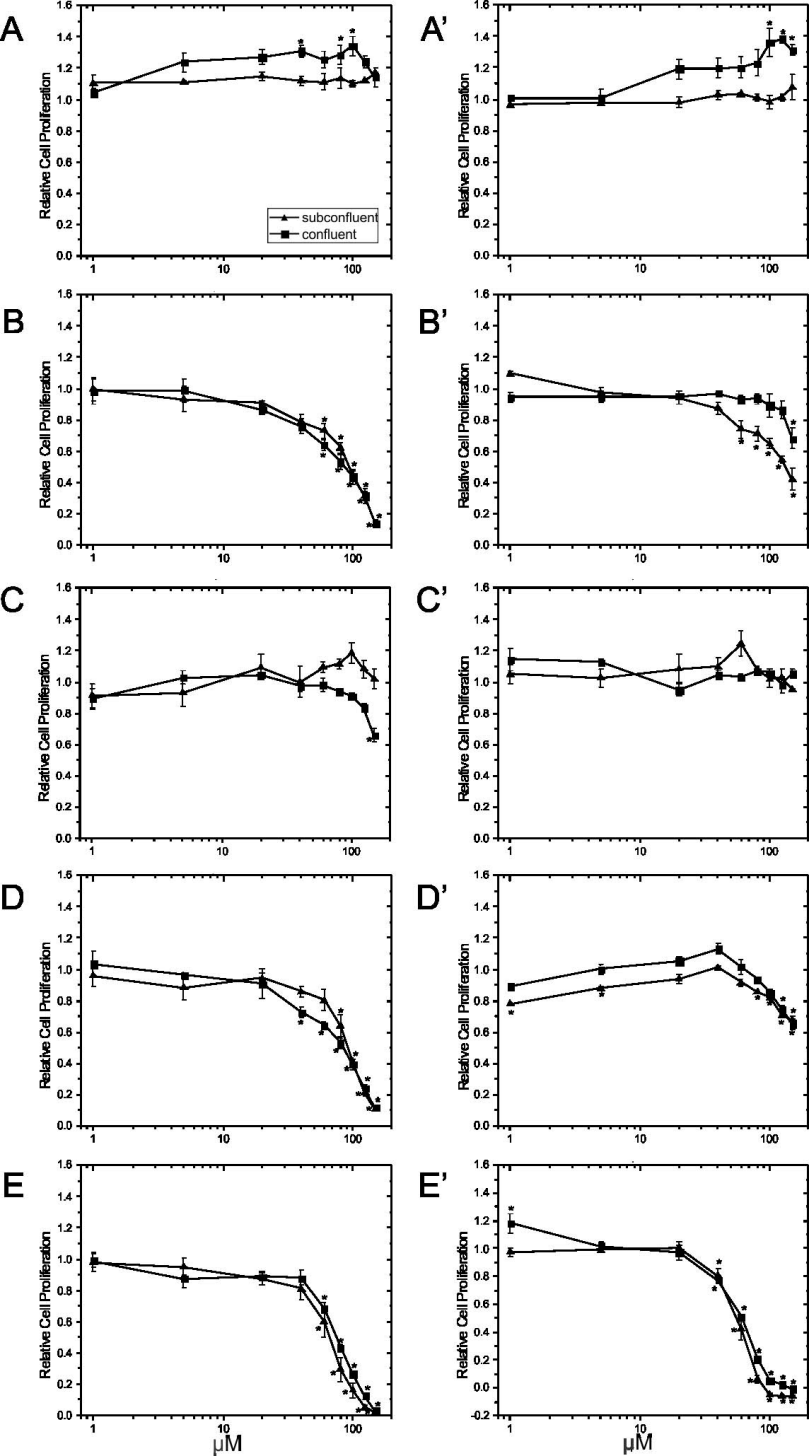
Effect of ECM substrate and growth state on DNA synthesis of EA.hy926 cells in presence of PUFAs. EA.hy926 endothelial cells were seeded on plastic culture plates at 9000 cells/cm^2^ in the absence (Panel A-E) or presence of Matrigel (Panel A’-E’). Growing cells (day 4) or confluent cells (day 8) were treated with different PUFAs [LA (Panel A, A’), AA (Panel B, B’), ALA (Panel C, C’), EPA (Panel D, D’) and DHA (Panel E, E’)] at final concentrations of 1, 5, 20, 40, 60, 80, 100, 125 and 150 μM for 24 hours, at which point, DNA synthesis was assessed using the BrdU cell proliferation assay. Data are normalized with vehicle control values and plotted as means ± SEM (n = 3). *Significantly different (p <0.05) from the vehicle control for the respective growth state.

PUFAs (final concentrations of 1, 5, 20, 40, 60, 80, 100, 125, and 150 μM) were added on day 4 to subconfluent and on day 8 to confluent cells grown without (Figs 8A–8E and 9A–9E) and with Matrigel (Figs 8A’–8E’ and 9A’–9E’). The cells were then incubated for 24 hours with the PUFA treatment, and viability (Fig 8) was determined with the CCK-8 kit. AA (Fig 8B), EPA (Fig 8D) and DHA (Fig 8E) decreased the viability of subconfluent cells grown without Matrigel in a dose-dependent manner, while LA (Fig 8A) and ALA (Fig 8C) had no negative effect. Compared to vehicle control, AA significantly decreased the viability of proliferating cells at 20 μM, while EPA and DHA began to reduce viability at 5 μM and 60 μM, respectively. In contrast, the viability of subconfluent cells when grown on Matrigel was unaffected by any of the PUFA treatments (Fig 8A’–8E’) except for DHA which reduced cell viability by about 20% at 125 and 150 μM. Although there were significant differences in viability observed at certain individual concentrations (ALA at 100 μM, AA at 80 and 125 μM in the depicted experiment for subconfluent cells), these effects were not consistently seen in replicate experiments.

The viability of confluent cells grown without Matrigel was generally unaffected by treatment with different PUFA except for LA (Fig 8A) and DHA (Fig 8E). Although viability was significantly decreased by LA at concentrations higher than 5 μM, the effect was small (-13%). DHA at 20 and 40 μM significantly increased the viability of confluent cells in the experiment shown, however, this was not observed in the replicate experiments. Viability of confluent cells grown on Matrigel was unaffected by treating the cells with different PUFAs except for ALA (Fig 8C’) and DHA (Fig 8E’). However, the differences detected for ALA at 60 and 80 μM were of small magnitude and not observed at higher concentrations or in replicate experiments. In contrast, DHA dose-dependently reduced cell viability by 12% and 30% at 125 μM and 150 μM, respectively, for confluent cells grown on Matrigel.

**Fig 10:**
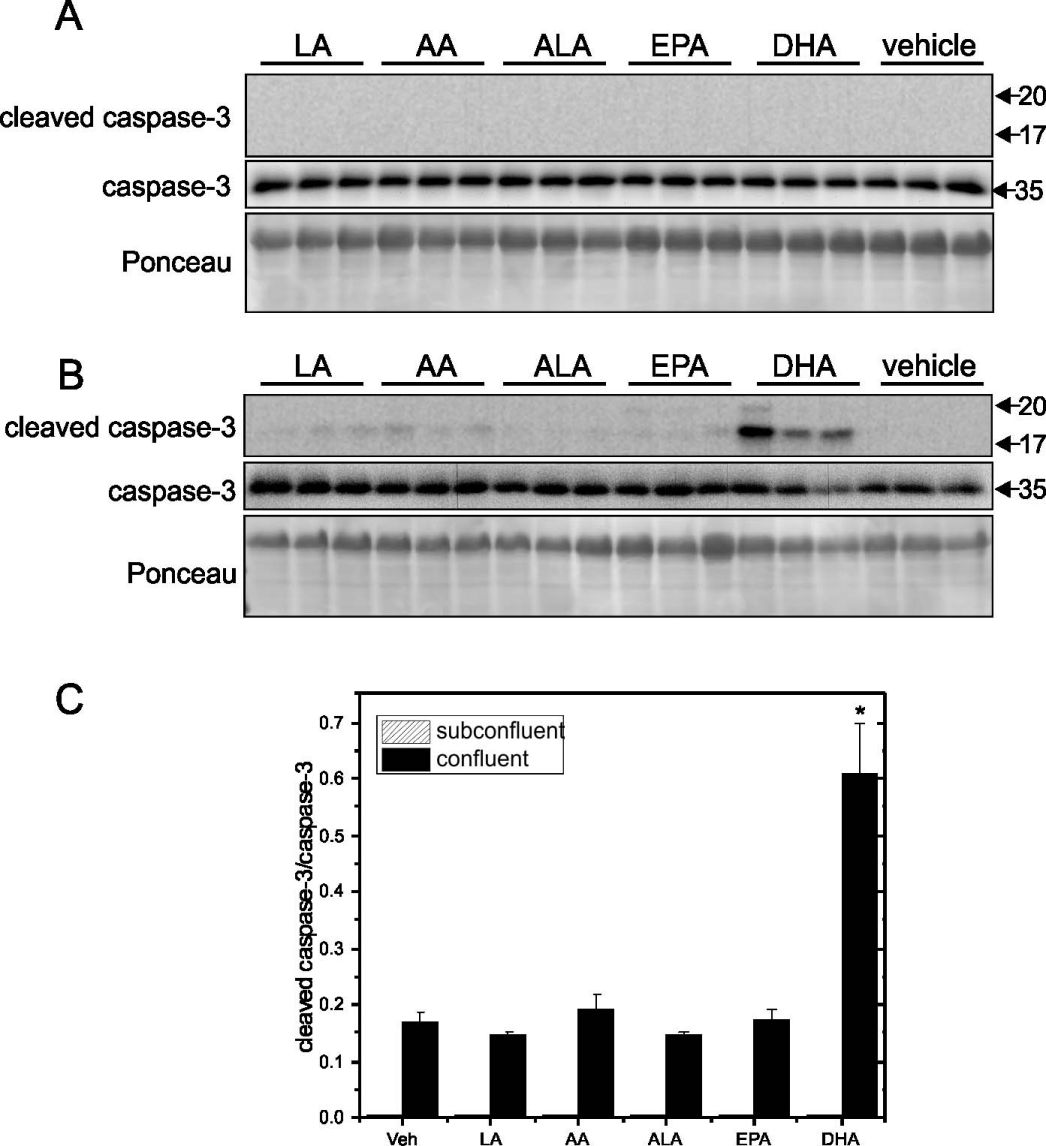
Effect of PUFA treatment on cleaved caspase-3 and caspase-3 in subconfluent and confluent EA.hy926 cells grown on Matrigel-coated plates. EA.hy926 endothelial cells were seeded on Matrigel-coated plates at 9000 cells/cm^2^ and grown for 4 days. Cells were treated with PUFAs (LA, AA, ALA, EPA and DHA) individually at 125 μM for 8 h. Protein levels of cleaved caspase-3 and caspase-3 were determined by Western blotting of cell lysates prepared after treatments. Representative blots are shown in Panel A (subconfluent) and Panel B (confluent). Densitometry was used to quantify the intensity of the bands in the upper panels. Data are presented as means ± SEM (n = 3) for cleaved caspase-3/caspase-3 (Panel C). *Significantly different (p <0.05) from vehicle control.

To examine how different PUFA treatments affect proteins associated with cell viability in relation to endothelial growth state, the relative levels of cleaved caspase-3 and caspase-3 were compared in subconfluent and confluent cells grown on Matrigel-coated plates (Fig 10A–10C). In subconfluent cells, there was no cleaved caspase-3 after various PUFA treatments suggesting the reduction of viability was not through a caspase-3 dependent pathway. In contrast, DHA significantly promoted caspase-3 cleavage in confluent cells indicating a higher level of active caspase-3.

When examining DNA synthesis, there were differences in the sensitivity of the cells in response to PUFAs. AA (Fig 9B and 9B’), EPA (Fig 9D and 9D’), and DHA (Fig 9E and 9E’) decreased the DNA synthesis rate of growing and confluent cells in a dose-dependent manner, while LA (Fig 9A and 9A’) and ALA (Fig 9C and 9C’) did not. Specifically, when cells were grown on Matrigel-coated plates, AA (Fig 9B’), EPA (Fig 9D’) and DHA (Fig 9E’) at concentrations higher than 60, 80, and 40 μM, respectively, decreased BrdU incorporation in growing cells, while EPA and DHA higher than 125 and 40 μM, respectively, reduced DNA synthesis in confluent cells. Interestingly, only the highest concentration of AA (150 μM) reduced DNA synthesis of confluent cells grown on Matrigel (Fig 9B’). On plastic culture plates, AA (Fig 9B), EPA (Fig 9D) and DHA (Fig 9E) at concentrations higher than 60, 80 and 60 μM, respectively, decreased BrdU incorporation in growing cells, while AA, EPA and DHA higher than 60, 40 and 60 μM, reduced DNA synthesis in confluent cells. It was also observed that treatment with the same concentrations of PUFA caused a greater reduction in DNA synthesis in the absence of Matrigel, especially for AA, EPA and DHA.

**Fig 11:**
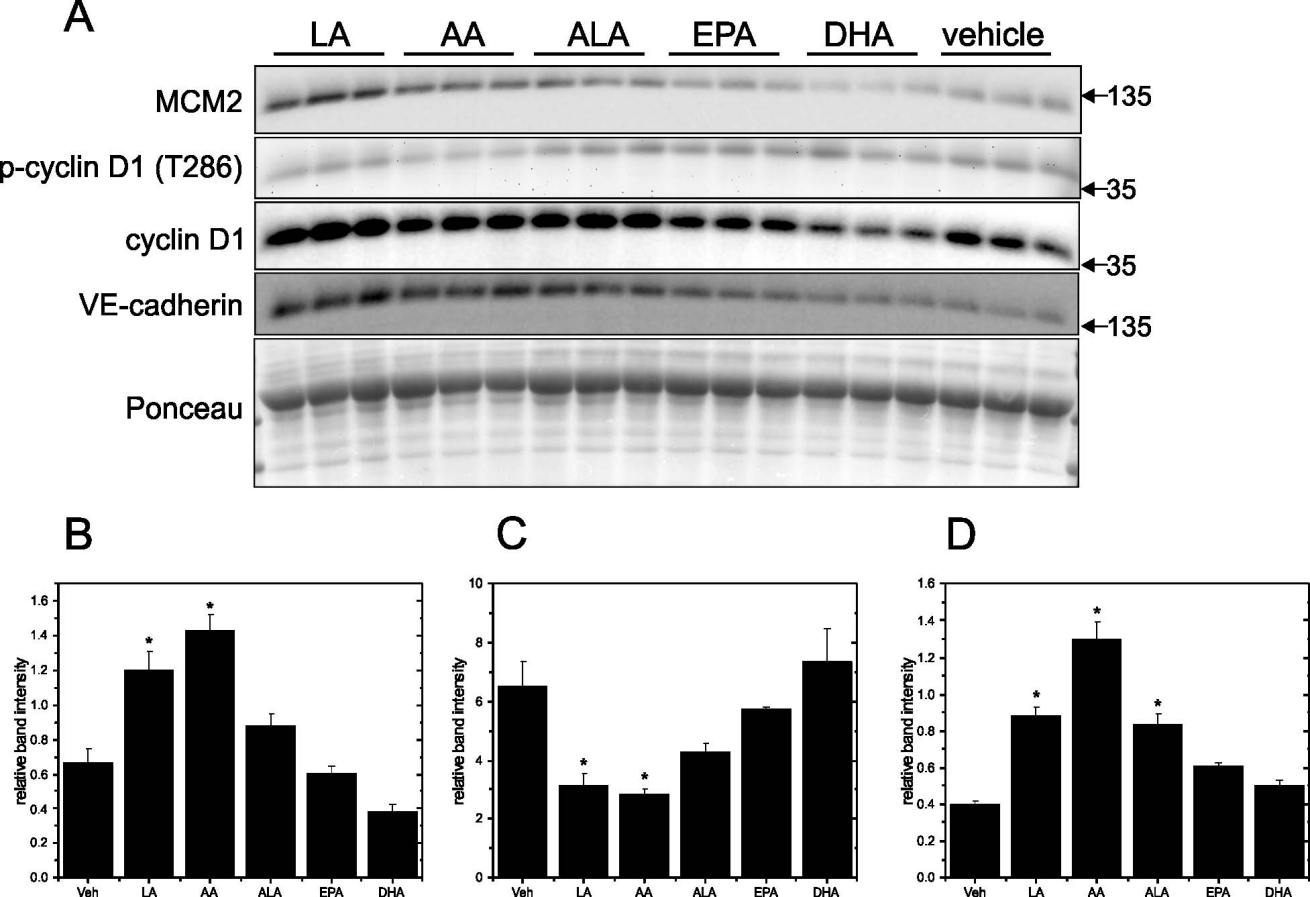
Effect of PUFA treatment on MCM2, p-cyclin D1, cyclin D1 and VE-cadherin in subconfluent EA.hy926 cells grown on Matrigel-coated plates. EA.hy926 endothelial cells were seeded on Matrigel-coated plates at 9000 cells/cm^2^ and grown for 4 days. Cells were treated with PUFAs (LA, AA, ALA, EPA and DHA) individually at 125 μM for 8 h. Protein levels of MCM2, p-cyclinD1, cyclin D1 and VE-cadherin were determined by Western blotting of cell lysates prepared after treatments. Representative blots are shown in Panel A. Densitometry was used to quantify the intensity of the bands in the upper panel and data were normalized to a band visualized by Ponceau staining. Data are presented as means ± SEM (n = 3) for MCM2 (Panel B), p-cyclin D1/cyclin D1 (Panel C) and VE-cadherin (Panel D). *Significantly different (p <0.05) from vehicle control.

**Fig 12:**
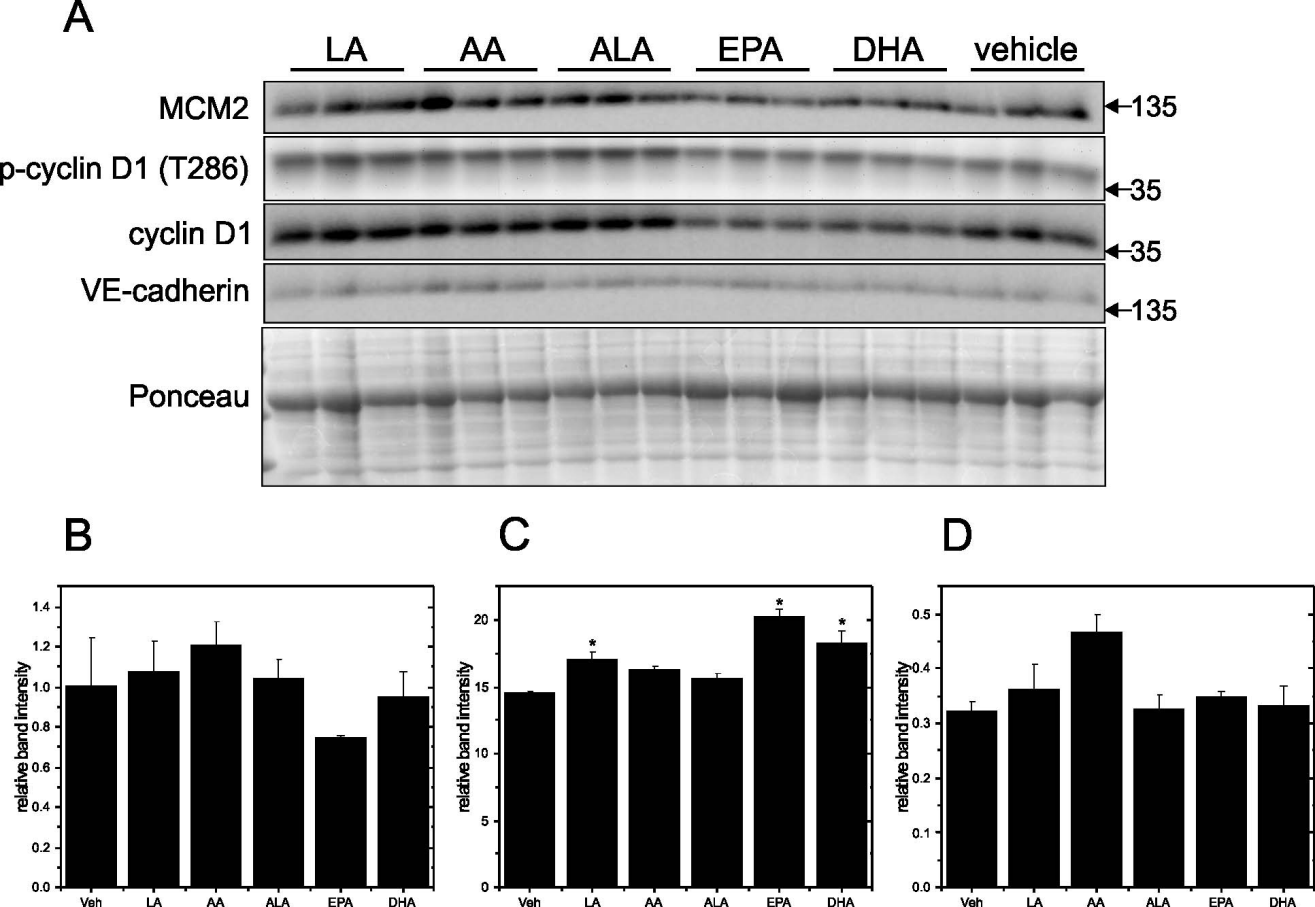
Effect of PUFA treatment on MCM2, p-cyclin D1, cyclin D1 and VE-cadherin in confluent EA.hy926 cells grown on Matrigel-coated plates. EA.hy926 endothelial cells were seeded on Matrigel-coated plates at 9000 cells/cm^2^ and grown for 8 days. Cells were treated with PUFAs (LA, AA, ALA, EPA and DHA) individually at 125 μM for 8 h. Protein levels of MCM2, p-cyclinD1, cyclin D1 and VE-cadherin were determined by Western blotting of cell lysates prepared after treatments. Representative blots are shown in Panel A. Densitometry was used to quantify the intensity of the bands in the upper panel and data were normalized to a band visualized by Ponceau staining. Data are presented as means ± SEM (n = 3) for MCM2 (Panel B), p-cyclin D1/cyclin D1 (Panel C) and VE-cadherin (Panel D). *Significantly different (p <0.05) from vehicle control.

To examine how different PUFA treatments affect proteins associated with cell proliferation and endothelial state, the relative levels of MCM2, p-cyclin D1 (T286), cyclin D1 and VE-cadherin were compared in subconfluent (Fig 11) and confluent cells (Fig 12) grown on Matrigel-coated plates. In subconfluent cells, LA and AA significantly increased MCM2 (Fig 11B) while decreasing the p-cyclin D1/cyclin D1 ratio (Fig 11C) compared to vehicle control. LA, AA and ALA also significantly increased VE-cadherin levels (Fig 11D). In confluent cells, LA, EPA and DHA significantly increased the p-cyclin D1 (T286)/cyclin D1 ratio (Fig 12C), but MCM2 (Fig 12B) and VE-cadherin (Fig 12D) were not changed by these PUFA treatments. These results support the conclusion that cells grown on Matrigel enter a quiescent state, while those cultured on plastic do not, even when mature cell junctions are present.

## Discussion

The present study is the first to show that EA.hy926 endothelial cells enter a quiescent state after reaching confluence when grown on Matrigel. VE-cadherin is important for maintaining and controlling of endothelial intercellular contacts, and its expression confirms the presence of cell junctions, which only occurs once a confluent monolayer is formed. This interpretation is possible because there is a decrease in the DNA synthesis rate as a result of contact inhibition and there is no loss of cell viability. Furthermore, there was a decline in MCM2 levels and the p-cyclin D1/cyclin D1 ratio, which indicates the cells had left the cell cycle. The high levels of p27kip1 across all time points supported the evidence that cell cycle progression to S phase is inhibited. In contrast, cells grown either without Matrigel or with collagen do not become quiescent, which is indicated by a continued high level of BrdU incorporation after confluence is reached. The high p-cyclin D1/cyclin D1 ratio and VE-cadherin levels with a low level of p27kip1 in cells grown on plastic also support the view the cells remain in the cell cycle even after cell-to-cell contacts have formed. Quiescent cells are also characterized by low levels of apoptosis and senescence. In the current study, cells were cultured for up to 12 days without changing the media. Accumulation of waste metabolites and deprivation of nutrients can lead to cell death due to apoptosis and cessation of growth due to senescence. However, a comparison of caspase-3 activity, which is an indicator for apoptosis, and SA-β-galactosidase activity, which is a known biomarker of senescent cells, has shown that cells grown on Matrigel have less potential to become either apoptotic or senescent. Thus, the evidence demonstrates that cells grown on Matrigel-coated plates are more likely to be quiescent than cells grown on plastic plates. Additionally, this study has shown that confluent cells are protected from high concentrations of PUFAs, since treatment with these compounds does not reduce viability regardless of whether they are grown on Matrigel or not. However, Matrigel promotes the viability of subconfluent cells exposed to PUFAs, specifically AA, EPA and DHA, while the precursor C18 LA and ALA have no negative effect on viability at even the highest concentration employed. Interestingly, DHA induced cleavage of caspase-3 in confluent cells but not subconfluent cells, which suggests that the effect of DHA on cell viability potentially involves an apoptotic mechanism that only operates in the quiescent state. Finally, AA, EPA and DHA have similar effects on BrdU incorporation in subconfluent and confluent cells, albeit with different sensitivities in relation to concentration, while the precursor PUFAs do not impact negatively on proliferation. Specifically, at higher concentrations, these PUFAs induce cell quiescence by suppressing DNA synthesis. Our results have thus established the critical importance of both ECM substrate and growth state in how endothelial cells respond to dietary factors such as PUFAs and have shown how specific PUFAs could potentially influence endothelial function in both healthy and pathological conditions when taken at a high dose such as in supplements.

Under physiological conditions, the endothelial cells present on the inner surface of blood vessels are in their resting, non-dividing state. However, endothelial cells have an intrinsic ability to exit this quiescent state and proliferate, which is typically seen in cell culture. But can these cells re-enter the quiescent state when they become confluent as they are in a healthy vessel? Our BrdU assay results showed that EA.hy926 endothelial cells become quiescent after confluence is reached as indicated by a decrease in the DNA synthesis rate, decrease of MCM2 expression and reduced p-cyclin D1/cyclin D1 ratio only when grown on a specific ECM substrate, namely Matrigel. Furthermore, the cell number data show that quiescence does not occur because of a loss of cell viability, thus implicating contact inhibition in the observed reduction of DNA synthesis. Interestingly, the cells do not become quiescent even after becoming confluent when grown on collagen-coated or uncoated plates. As a result, our study is the first to show that a thin layer coating of Matrigel provides better cellular attachment, thus enabling entry into a quiescent state *in vitro*, which is a more representative model of endothelial cell characteristics and responses *in vivo*. At the same time, it is noteworthy that no study has used Matrigel as an ECM substrate to grow EA.hy926 endothelial cells and we have demonstrated its presence protects the viability of cells against the effects of higher concentrations of PUFAs (AA, EPA and DHA). Although a few studies have used other ECM substrates such as gelatin [13–15] and fibronectin [16] to test the viability of endothelial cells in response to PUFA treatment, comparisons with non-coated plates were lacking.

The importance of growth state has received limited attention with respect to the endothelial cell response to PUFAs. We have shown that confluent cells have higher viability than growing cells in the presence of higher concentrations of PUFAs, specifically AA, EPA and DHA. In support of our findings, the viability of confluent HUVECs [17], confluent primary porcine pulmonary artery endothelial cells [13], confluent primary human saphenous vein endothelial cells [16], and confluent and post-confluent EA.hy926 endothelial cells [18] were not affected by treatment with various PUFAs, while the viability of proliferating HUVECs [14, 17, 18] was reduced. There are two studies showing that viability of 70–90% confluent EA.hy926 [19] and HUVECs [20] was not affected by treatment with 100 μM DHA for 24 h and 8 h, respectively. Although the cells were not 100% confluent, the establishment of cellular contact as seen in cell cultures nearing confluency is still sufficient to help survival. Studies that did not indicate the growth state of the endothelial cells are excluded from the discussions here [21–23].

The range of fatty acid concentrations in the present study was chosen to be representative of the circulating free fatty acid concentrations that would be in contact with the endothelium *in vivo* [3]. We observed that viability and proliferation of growing endothelial cells was more sensitive to C20 and C22 PUFAs (AA, EPA and DHA) than their precursor PUFAs (LA and ALA, which are the two dietary essential fatty acids), regardless of whether they are omega-6 or omega-3 fatty acids. Cell viability was decreased when AA was >20 μM, EPA >5 μM, and DHA > 60 μM in growing endothelial cells in the absence of Matrigel, whereas 150 μM of LA or ALA for 24 h had no effect on viability. Likewise, Spector et al [24] reported that treatment with LA (150 μM for 6 h) does not affect the viability of growing primary HUVECs, while Kim et al [17] found that that 40 μM DHA induced death in growing cells in a time-dependent manner. Gousset-Dupont et al [18] reported 40 μM of EPA and DHA reduced viability of growing EA.hy926 endothelial cells, which compares favourably with our observations that EPA and DHA were lethal to cells at 5 μM and 60 μM, respectively. Concentrations higher than 150 μM and incubation times longer than 24 h were not tested in our experiments, but in other studies 300 μM LA or AA for 24 hours induced apoptosis of growing primary HUVECs, with further increases in apoptosis when the incubation time was extended to 48 h [14]. We did not examine FA concentrations above 150 μM since0020030030030030total non-esterified FA levels in plasma are approximately 300 μM in healthy individuals [3]. Of that total, LA represents approximately 25% [25] or about 75 μM. Furthermore, LA is substantially higher than the other PUFAs, which range from 3–8 μM in their non-esterified form [25].

In the present study, DNA synthesis of both growing and confluent cells grown on Matrigel-coated and plastic plates was reduced in a dose-dependent manner, particularly by the elongated PUFAs (AA, EPA, DHA). Interestingly, AA slightly lowered the DNA synthesis rate of confluent cells grown on Matrigel-coated plates but only at the highest concentration (150 μM) tested in these cultures. The data presented by Kim et al [17] are in agreement with our results showing 40 μM DHA decreased proliferation of growing HUVECs after 24 h, while 40 μM AA did not affect proliferation of either growing or confluent cells. However, Kim et al [17] also showed that 40 μM DHA did not decrease the proliferation of confluent HUVECs after 24 h, whereas we observed the opposite. On the other hand, Artwohl et al [14] showed that treatment of growing HUVECs with 300 μM LA and AA for 48 h caused cell cycle arrest. Although their experimental conditions (concentration and time length) went beyond what we employed, we did find that, after 24 hours, 150 μM of LA did not affect proliferation while AA at concentrations higher than 60 μM decreased DNA synthesis in growing cells. Although absolute uniformity between these studies does not exist, it is apparent that certain concentrations of DHA and AA do consistently slow the proliferation rate of growing cells.

We have also explored potential mechanisms of how different PUFAs affect the viability and proliferation of growing and confluent cells by comparing specific proteins associated with cell apoptosis including cleaved caspase-3 and caspase-3, and proteins associated with proliferation (MCM2, p-cyclin D1, cyclin D1) as well as VE-cadherin, which is an endothelial-specific junction molecule important in establishing contact inhibition and thus controlling proliferation [26]. We observed an increase of cleaved caspase-3 in confluent cells grown on Matrigel by DHA but not other PUFAs, while active caspase-3 was not seen in subconfluent cells. This suggests that the reduction in viability seen with DHA treatment of both growing and confluent cells occurs by different mechanisms. Furthermore, this is the first time activation of cleaved caspase-3 has been reported in confluent human endothelial cells in response to direct DHA treatment. Artwohl et al [27] found caspase-8 activation is associated with apoptosis induced by 300 μM of LA, AA and ALA on HUVECs, human aortic endothelial cells, human retinal endothelial cells and endothelial progenitor cells. Artwohl [14] also found 300 μM of LA and AA induced apoptosis of HUVECs by reducing apoptosis inhibitor bcl-2 and increasing levels of bak expression. Meerarani et al [28] have reported an up-regulation of caspase-3 by 90 μM LA on porcine pulmonary arterial endothelial cells. In terms of the changes of proliferation related molecules by different PUFAs, our results indicate that indirect modulation of the cellular machinery that regulates cell proliferation by AA, EPA and DHA treatment is the reason these PUFAs reduce DNA synthesis. However, other possible mechanisms were not examined in this study. Few studies have looked at these molecules when investigating the effect of PUFAs on endothelial cell proliferation. Su et al [29] showed high concentrations of free fatty acids affect the expression of cell cycle-related proteins such as cyclin B, cyclin D, cyclin E, p53, and cell division cycle protein 14 in human brain vascular endothelial cells, although the fatty acid composition of their treatment was not specified. Artwohl et al [14] showed a correlation between an increase in the expression of p21 (inhibitor of cyclin-dependent kinase), a decrease of cyclin D3 protein expression, an increased number of cells in G_0_/G_1_ phase, and a decreased number of cells in S phase when they reported LA and AA caused cell cycle arrest in HUVECs, and suggested a sophisticated cell cycle controlling mechanisms. When Kim et al [17] reported exposure for 24 h with 40 μM DHA decreased the proliferation of HUVECs, they also indicated there was an increase of cells in the sub G_1_ phase. Given this uncertainty in how they operate, the mechanisms by which AA, EPA and DHA reduce DNA synthesis still require further investigation.

Cell preparation before treatment may be a factor affecting the cellular response to PUFAs. We have shown that up to 60 μM AA does not increase proliferation and may slightly decrease proliferation of subconfluent cells. On the other hand, Fiorio et al [15] showed that adding 5 μM AA to growing breast lobular-infiltrating carcinoma (B-TEC) derived endothelial cells for 24 hours increased proliferation (according to cell number) compared to control, and that 2 μM AA significantly increased the number of bovine aortic endothelial cells compared to 5% FBS alone [30]. It is worth pointing out that in the studies by Fiorio et al [15, 30], cells were starved in the presence of low serum concentration (1%) for 24 h before adding AA. Given this experimental design, AA could be stimulating the proliferation of serum-starved cells. We believe that adding PUFAs to the original media instead of to pre-starved cells has greater physiological relevance.

Studies on how PUFAs can modulate endothelial function are of great importance because on one hand, healthy endothelial cells are vital in maintaining cardiovascular homeostasis while on the other hand, endothelial dysfunction promotes the development and progression of vascular disease [31]. Dietary PUFAs could modulate various aspects of vascular function in both healthy and dysfunctional endothelial cells. Consumption of n-3 PUFA is associated with reduced cardiovascular disease risk [32] and it is proposed that the beneficial effects of n-3 PUFA may be due to their effects on endothelial cell function [33]. Our study is the first to show how different PUFAs, including n-6 PUFA (LA and AA) and n-3 PUFA (ALA, EPA and DHA), impact endothelial cells differently in terms of viability and proliferation. Further studies are still required to determine the effects of PUFAs on other functions of endothelial cells such as migration and barrier function.

In conclusion, our results have shown that extracellular matrix proteins, which can modulate growth state, are important for the EA.hy926 endothelial cell response to different PUFAs in terms of viability and proliferation. This observation may also apply to other dietary factors, but studies examining this possibility have not been done. Higher concentrations of PUFA, especially AA (>20 μM), EPA (>5 μM) and DHA (>60 μM), reduce cell viability, particularly when the cells are not grown on an ECM substrate. However, confluent endothelial cells that become quiescent due to contact inhibition, which is the state that represents the normal endothelial cell monolayer in a healthy vessel, are more resistance to high concentrations of PUFAs than subconfluent, growing cells. Another unique finding of this study is DHA treatment significantly induced cleaved caspaes-3 in quiescent endothelial cells, which are representative of the normal healthy endothelium. Further investigation is therefore warranted to determine the clinical significance of this observation as it applies to the physiological and pathological conditions of endothelial cells in the vasculature. We have also shown that AA, EPA and DHA, but not their precursor fatty acids (LA and ALA), reduce DNA synthesis of EA.hy926 endothelial cells grown on Matrigel-coated and plastic plates in a dose-dependent manner. However, whether these responses to PUFAs are favourable or detrimental to endothelial cells and overall vascular function will depend on whether cells are healthy or dysfunctional, and these conditions warrant further investigation.
